# Comparison of liver exposure in CT-guided high-dose rate (HDR) interstitial brachytherapy versus SBRT in hepatocellular carcinoma

**DOI:** 10.1186/s13014-021-01812-7

**Published:** 2021-05-06

**Authors:** Franziska Walter, Lukas Nierer, Maya Rottler, Anna Sophie Duque, Helmut Weingandt, Justus Well, Roel Shpani, Guillaume Landry, Max Seidensticker, Florian Streitparth, Jens Ricke, Claus Belka, Stefanie Corradini

**Affiliations:** 1 Department of Radiation Oncology, University Hospital, LMU Munich, 81377 Munich, Germany; 2 Department of Radiology, University Hospital, LMU Munich, Munich, Germany

**Keywords:** CT-guided interstitial brachytherapy, Stereotactic body radiotherapy, Hepatocellular carcinoma

## Abstract

**Background:**

In unresectable hepatocellular carcinoma several local ablative treatments are available. Among others, radiation based treatments such as stereotactic body radiotherapy (SBRT) and high-dose rate interstitial brachytherapy (HDR BT) have shown good local control rates.

**Methods:**

We conducted a dose comparison between actually performed HDR BT versus virtually planned SBRT to evaluate the respective clinically relevant radiation exposure to uninvolved liver tissue. Moreover, dose coverage and conformity indices were assessed.

**Results:**

Overall, 46 treatment sessions (71 lesions, 38 patients) were evaluated. HDR BT was applied in a single fraction with a dose prescription of 1 × 15 Gy. D98 was 17.9 ± 1.3 Gy, D50 was 41.8 ± 8.1 Gy. The SBRT was planned with a prescribed dose of 3 × 12.5 Gy (65%-Isodose), D98 was 50.7 ± 3.1 Gy, D2 was 57.0 ± 2.3 Gy, and D50 was 55.2 ± 2.3 Gy.

Regarding liver exposure Vliver10Gy_BT_ was compared to Vliver15.9Gy_SBRT_, Vliver16.2Gy_SBRT_ (EQD2 equivalent doses), and Vliver20Gy_SBRT_ (clinically relevant dose), all results showed significant differences (*p* < .001). In a case by case analysis Vliver10Gy_BT_ was smaller than Vliver20Gy_SBRT_ in 38/46 cases (83%). Dmean of the liver was significantly smaller in BT compared to SBRT (*p* < .001).

GTV volume was correlated to the liver exposure and showed an advantage of HDR BT over SBRT in comparison of clinically relevant doses, and for EQD2 equivalent doses. The advantage was more pronounced for greater liver lesions The Conformity Index (CI) was significantly better for BT, while Healthy Tissue Conformity Index (HTCI) and Conformation Number (CN) showed an advantage for SBRT (*p* < .001).

**Conclusion:**

HDR BT can be advantageous in respect of sparing of normal liver tissue as compared to SBRT, while providing excellent target conformity.

## Introduction

The treatment of hepatocellular carcinoma (HCC) is challenging and today there is a wide range of effective treatment options available for the respective stage of disease [1]. Therefore, decision making on the optimal treatment strategy for individual patients is complex and usually made in interdisciplinary conferences. Treatment of early stage disease with curative intend typically involves either surgical resection or locally ablative treatments (LAT), and liver transplantation (LT). With only 5–10% of patients as suitable candidates for surgical resection [2] there is a high demand for treatment strategies in non-surgical early stage HCC patients. Various LAT are available nowadays with different modes of action such as radiofrequency ablation (RFA) or microwave ablation. Radiation-based LATs such as stereotactic body radiation therapy (SBRT) and high-dose-rate brachytherapy (HDR BT) have evolved in recent years as alternative or as multimodality treatment in combination with other conventional LAT options. In a randomized phase II trial radiation based treatment of HCC even showed favourable time to progression rates compared to conventional transarterial embolization [3]. HDR BT therefore has been adopted as treatment option in early stage HCC by the ESMO guidelines [1]. Liver SBRT has been shown to be a safe and effective treatment option in the definitive setting or as a bridging treatment for patients awaiting LT [4]. It is typically administered in 3–6 fractions with a dose per fraction ranging from 8 to 20 Gy [5]. For target volume motion management a dedicated 4D-CT with intravenous (IV) contrast is necessary and in many cases abdominal compression or respiratory gating is required. For conventional linear accelerator-based SBRTs, either invasively placed fiducial markers, surgical clips from prior surgery or contrast enhancement of lipiodol following transcatheter arterial chemoembolization are utilized. The emerging technique of MR-guided radiotherapy allows for omission of implanted markers since direct visualization of liver tumours and treatment adaption is possible [6, 7].

Similarly, single fraction HDR BT is a highly effective local treatment [8] with several features desirable for LAT. Unlike thermal ablation, HDR BT is not subject to the cooling-effect of adjacent great vessels and can therefore be applied in centrally located lesions without loss of efficacy. While RFA is technically restricted to smaller lesions and spherical shapes, HDR BT can also cover larger and more irregular shaped targets depending on the number of catheter used for the implant. Despite these advantages both SBRT and more so BT are scarcely utilized in patients with HCC and underlying liver cirrhosis due to concerns of treatment-induced liver damage.

Independent of the treatment modality the post-interventional liver function is predictive for patient survival [9]. In case of radiation-based treatment overall exposure of uninvolved liver tissue and risk of radiation induced liver disease (RILD) need to be considered by the treating physician. Functional MRI adequately detects post-interventional changes in liver tissue.

Following SBRT, morphological changes on diagnostic imaging such as CT [10] or MRI [11] have been reported. More specifically, changes in signal intensity in T1 and T2-weighted MRI sequences were shown to correlate with isodoses > 20 Gy (T1: 21.9 ± 6.7 Gy T2: 22.4 ± 6.6 Gy) [12]. Similarly, radiation-induced liver damage after interstitial HDR BT was detected on MRI in liver tissue exposed to > 10 Gy which was correlated with histological specimens [13–15].

The objective of the current study was to compare features of radiation-based local ablative treatments. We therefore conducted a planning study using planning CT datasets acquired for interstitial BT in patients with maximal 3 HCC lesions. Patients were treated with BT in a single fraction with 15 Gy prescribed to D100. The comparison was done with SBRT in three fractions with 37.5 Gy (normalisation to 65%-isodose) which is within the range of published data [16]. The primary endpoint of this planning study was to compare normal liver exposure in single fraction interstitial BT versus fractionated SBRT. Secondary endpoints were comparison of dose coverage and conformity indices.

## Patients and methods

We reviewed all patients who were treated for a HCC with single-fraction HDR interstitial brachytherapy in our institution between 07/17 and 02/19. For this analysis we included treatment sessions with up to 3 HCC lesions and a maximum diameter of 6 cm as this would be patients eligible for either SBRT or HDR BT. 46 treatment plans were included in this retrospective planning study. All patients had a good liver function, and a Child–Pugh score of A or B.

### HDR BT planning and application

The brachytherapy catheter were placed under CT-guidance by an interventional radiologist. All details regarding the procedure have been described elsewhere [17]. With the BT catheters in place, a planning CT was acquired in expiration breath hold with a slice thickness of 2 mm. IV contrast was administered in 24 cases. The planning CT was transferred to the treatment planning software (TPS) Oncentra Brachy (Elekta AB, Stockholm, Sweden) version 4.5.2. Total liver and adjacent organs at risks (OAR) such as duodenum, stomach, bowel or kidney were contoured. The clinical target volume (CTV) was defined by a radiation oncologist on the planning CT with the use of additional pre-treatment diagnostic imaging (CT with IV contrast or liver specific MRI with hepatocyte specific contrast). Since no setup margin is necessary in interstitial BT, the planning target volume for brachytherapy (PTV_BT_) was identical to the CTV. Catheter reconstruction and dose optimization were performed by a medical physicist. The hyperdense catheter tip markers of the BT catheters (Primed Halberstadt Medizintechnik, Halberstadt, Germany) were used as reference points for the 3D catheter reconstruction on the planning CT. Dwell point step width was 2 mm, and high PTV_BT_ coverage and OAR sparing with soft dwell time modulation restrictions [10] was sought. Dose plan optimization was carried out manually and in some cases using hybrid inverse treatment planning optimization (HIPO) [18] as a starting point for manual optimization. Oncentra Brachy v. 4.5.2. utilizes dose calculation according to the AAPM TG‐43U1 formalism. Of note, TG-43U1 has been shown to overestimate dose to liver tissue [19] compare to Monte Carlo dose calculation.

The prescribed dose was 15 Gy administered to 100% of the target volume (PTV_BT_ D100). Dose constraints for organs at risk were applied as given in Table 1. Maximal diameter and volume of PTV_BT_, and liver volume were extracted from the TPS. The administered doses to the PTV_BT_ D100, D98 and D50 were reported for comparison to SBRT plans. The D2 could not be reported for technical reasons (maximum dose close to catheter in BT and above the TPS dose reporting limits).

**Table 1:** Organs at risk: dose constraints

Organ	DVH value	Dose [Gy]
*BT*		
Bowel	D1 ccm	12
	D0.1 ccm	15
Colon	D1 ccm	12
	D0.1 ccm	15
Stomach	D1 ccm	12
	D0.1 ccm	15
Esophagus	D1 ccm	15
	D0.1 ccm	18
Myelon	D1 ccm	10
	D0.1 ccm	12
Liver hilum	D1 ccm	18
	D0.1 ccm	20
Skin	D1 ccm	10
*Sbrt*		
Liver	Mean dose	13
	D700 ccm	15
Stomach	Dmax	22
	D10 ccm	16.5
Duodenum	Dmax	22
	D5 ccm	16.5
Myelon	Dmax	18
Kidney	D200 ccm	14.5

### Virtual SBRT planning

Using the planning CT datasets acquired for BT, SBRT was planned retrospectively using the TPS Oncentra External Beam (Elekta AB, Stockholm, Sweden) version 4.5.2. for an Elekta Synergy linac with Agility multileaf collimator. 12–17 beams (6MV flattened beam) per SBRT plan and lesion were applied using conventional 3D conformal planning.

The same CTV was used as for BT, but it was expanded by 6 mm isotropically to create a Planning Target Volume SBRT (PTV_SBRT_) according to our clinical practice. Since no information on breathing motion was available, no internal target volume (ITV) concept was used.

The prescribed dose was 37.5 Gy (normalisation to 65%-isodose) in three fractions [20]. The dose constraints for organs at risk are given in Table 1. The volume of PTV_SBRT_ and administered doses to the CTV D98, D50, D2 were reported and compared to BT dose coverage.

### Evaluation of liver exposure

We report the mean liver exposure for each treatment session (Dmean_liver_BT_). For evaluation of the liver exposure in HDR BT, ROIs were created using the 5 Gy and the 10 Gy-isolines, their total volumes are reported as V5Gy_BT_ and V10Gy_BT_. Intersections were applied with the liver contour to quantify the liver volume exposed to 5 Gy or 10 Gy as Vliver5Gy and Vliver10Gy. We applied the linear quadratic model to calculate the respective EQD2 for α/β 3 and 2. As reported by Seidensticker et al. the 10 Gy liver exposure determines radiation-induces liver damage that presents on MRI with hepatocyte specific contrast media and in histological specimen [13–15]. We therefore defined the 10 Gy liver exposure (volume) as clinically relevant liver exposure in single fraction HDR BT.

Regarding SBRT plans, the 20 Gy liver exposure in fractionated SBRT has been reported to exhibit changes on MRI [12] similarly to the 10 Gy exposure in a single fraction HDR BT. We therefore defined the 20 Gy exposure (volume) as clinically relevant liver exposure for SBRT. To assess the liver exposure in SBRT plans we created the following ROIs: V15.9Gy_SBRT_, V16.2Gy_SBRT_, and V20Gy_SBRT_. Intersections with the liver contour were applied to create Vliver15.9Gy_SBRT_, Vliver16.2Gy_SBRT_, and Vliver20Gy_SBRT_. To compare the two treatment modalities, we statistically compared the Vliver10Gy_BT_ with the corresponding clinically relevant dose Vliver20Gy_SBRT_, as well as with the corresponding equivalent doses to a single fraction 10 Gy liver exposure for different α/β values (EQD2; assuming α/β = 3 Gy [26 Gy] and α/β = 2 Gy [30 Gy]): Vliver15.9Gy_SBRT_ and Vliver16.2Gy_SBRT_ according to the linear-quadratic model calculated for 3 SBRT fractions (see also Table 4). The Dmean_liver_SBRT_ was also reported.

### Correlation of lesion volume and liver exposure

To assess the correlation of GTV volume and liver exposure we plotted all lesion GTV volumes against their corresponding liver exposure volumes and performed a second degree polynomial fit to the data points using the least squares method.

### Evaluation of conformity

The Conformity Index (CI) [21] defined as the proportion of PTV receiving at least the prescribed dose (PD) was calculated with V_PTV PD_ defined as volume (V) of PTV covered with the prescribed dose (PD), and V_PTV_ defined as volume of the PTV (0 ≤ CI ≤ 1; ideally 1).

$${\text{CI}} = \frac{{V_{{{\text{PTV}} {\text{PD}}}} }}{{V_{{{\text{PTV}}}} }}$$

The Healthy Tissue Conformity Index (HTCI) [21] indicates the irradiation of healthy tissue beyond the PTV border with the prescribed dose. V_PD_ was defined as the total volume receiving the prescribed dose (0 ≤ HTCI ≤ 1; ideally 1).

$${\text{HTCI}} = \frac{{V_{{\text{PTV PD}}} }}{{V_{{{\text{PD}}}} }}$$

The Conformation Number (CN) [21] incorporates PTV coverage and sparing of normal tissue (0 ≤ CN ≤ 1; ideally 1).

$${\text{CN}} = {\text{CI}} \cdot {\text{HTCI}} = \frac{{\left( {{\text{V}}_{{\text{PTV PD}}} } \right)^{2}}}{{{\text{V}}_{{{\text{PTV}}}} \cdot {\text{V}}_{{{\text{PD}}}} }}$$

### Statistical analysis

Statistical analysis was performed using the Statistical Package for Social Sciences (SPSS, Version 25, SPSS Inc, Chicago, IL). The analysis of patient related data was descriptive. The volumes of liver exposure V10liverGy_BT_ was compared with Vliver20Gy_SBRT_, Vliver16.2Gy_SBRT_, and Vliver15.9Gy_SBRT_ using the paired Wilcoxon signed-rank test, a *p* value < 0.05 was considered significant. Analogous, Dmean_liver_BT_ was compared to Dmean_liver_SBRT_, and the respective values of CI, HTCI, and CN were compared between BT and SBRT.

The study was performed in accordance to the declaration of Helsinki in its latest version and was approved by the Ethics Committee, LMU Munich (No. 18-511). The study was exempt from obtaining written informed consent due to its retrospective character. For this dose planning study, all patient data were anonymized irreversibly, no clinical follow-up data was obtained.

## Results

A total of 46 treatment sessions in 38 patients undergoing interstitial BT were included in this retrospective planning study. The patients were 32 male and 6 female, and the mean age was 67 ± 12 years. The Child–Pugh score prior to treatment was determined as Child A in 39 cases and Child B in 7 cases at the time of BT (Table 2). Overall, 27 patients had received prior treatments for HCC such as resection, RFA, TACE or systemic treatment. Three patients subsequently underwent liver transplant after HDR BT.

**Table 2:** Treatment and patient characteristics

*Gender*	
Male	32
Female	6
Age	67 ± 12 years
*Child–pugh-score*	
Child A	39
5 points	27
6 points	12
Child B	7
7 points	2
8 points	4
9 points	1
*Treatment volumes*	
1	25
2	17
3	4
*Number of BT catheter*	
1	17
2	18
3	8
4	3
*PTV*_*BT*_	
Diameter	2.93 ± 1.01 cm
Volume	9.91 ± 12.90 ccm
*PTV*_*SBRT*_	
Volume	31.03 ± 28.40 ccm
*Liver*	
Volume	1637.02 ± 492.58 ccm

Regarding the number of lesions, in 25 cases a single lesion was treated, in 17 cases two lesions, and in 4 cases three lesions were treated in one treatment session. The mean liver volume was 1637 ± 492 ccm, the mean diameter of HCC lesions was 2.9 ± 1.0 cm.

### Plan evaluation HDR BT versus SBRT

A total of 71 HCC lesions were treated with HDR BT. The mean PTV_BT_ was 9.9 ± 12.9 ccm. The HDR BT prescription was 15 Gy to D100 of the PTV_BT_ and the applied D100 was 15.0 ± 1.0 Gy. The near-minimum dose D98 was 17.9 ± 1.3 Gy, near-maximum dose D2 could not be evaluated, and the D50 was 41.8 ± 8.1 Gy. All OAR constraints were met. Table 3 gives an overview on the dose coverage.

**Table 3:** Target volume (CTV) dose coverage

	Dose [Gy]
*BT*	
D100	15.0 ± 1.0
D98	17.9 ± 1.3
D50	41.8 ± 8.1
D2	na
*SBRT*	
D98	50.7 ± 3.1
D50	55.2 ± 2.3
D2	57.0 ± 2.3

Correspondingly, for 71 HCC lesions SBRT plans were virtually planned. The mean PTV_SBRT_ was 31.0 ± 28.5 ccm. All OAR constraints were met. In SBRT plans, the prescribed dose to the PTV_SBRT_ was 37.5 Gy in 3 fractions to the 65%-Isodose, near-minimum dose D98 was 50.7 ± 3.1 Gy, near-maximum dose was 57.0 ± 2.3 Gy, and D50% was 55.2 ± 2.3 Gy (Table 3).

### Liver exposure

**Fig. 1:**
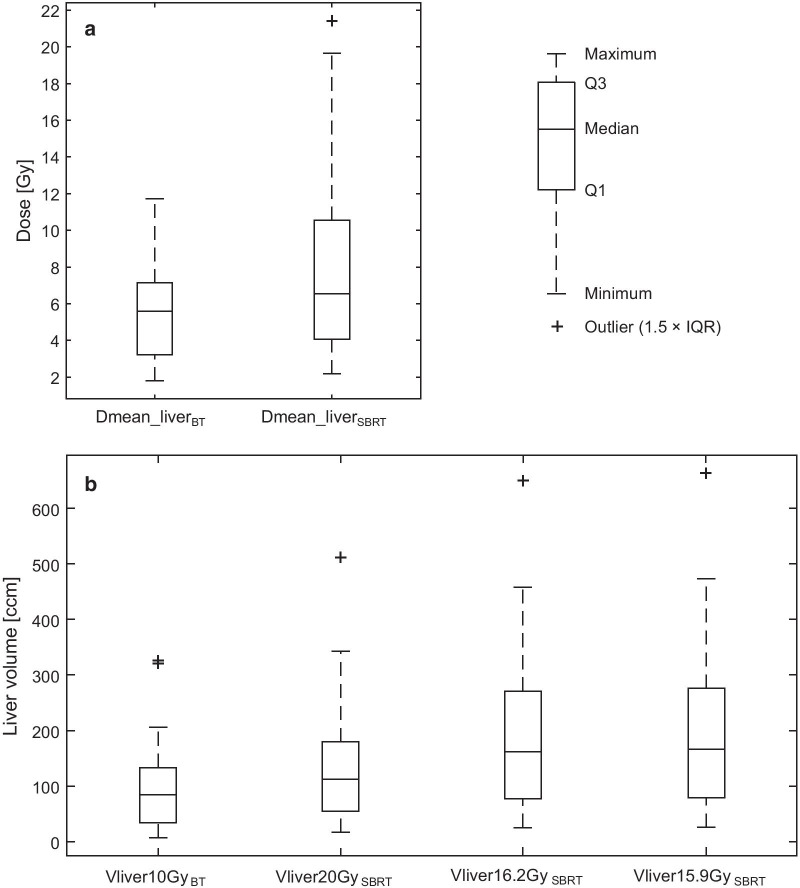
Liver exposure. **a** Overall liver exposure in Dmean **b** Liver exposure of 10 Gy in BT versus 20 Gy, 16.3 Gy, and 15.9 Gy in SBRT

Concerning liver exposure, the volume that received Vliver5Gy_BT_ and Vliver10Gy_BT_ in HDR BT was 219.3 ± 170.1 ccm and 92.8 ± 73.1 ccm, respectively. In SBRT, the liver exposure to Vliver15.9Gy_SBRT_, Vliver16.2Gy_SBRT_ and Vliver20Gy_SBRT_ was 186.4 ± 132.2 ccm, 181.5 ± 129.1 ccm and 134.5 ± 99.6 ccm, respectively (Table 4). When Vliver10Gy_BT_ was compared to Vliver15.9Gy_SBRT_, Vliver16.2Gy_SBRT_ and Vliver20Gy_SBRT_, results were all significantly different (*p* < 0.001) (Fig. 1b). In a case by case analysis, liver dose exposure with 10 Gy in HDR BT was usually smaller than irradiated liver volumes in SBRT (20 Gy) in 38/46 cases (83%). Dmean of the liver was significantly smaller in BT compared to SBRT (*p* < 0.001).

**Table 4:** Liver exposure

Technique	Dose [Gy]	EQD2 (α/β = 3 Gy) [Gy]	EQD2 (α/β = 2 Gy) [Gy]	Volume [ccm]	Liver volume [ccm]
*BT*					
	5.0	8.0	8.8	320.4 ± 244.2	219.3 ± 170.1
	10.0	**26.0**	**30.0**	109.1 ± 86.1	92.8 ± 73.1
*SBRT*					
	15.9	**26.4**	29.0	287.6 ± 206.0	186.4 ± 132.2
	16.2	27.2	**30.0**	276.9 ± 198.8	181.4 ± 129.1
	20	38.7	43.4	182.3 ± 134.2	134.5 ± 99.6

EQD2 equivalent doses marked in bold

### Correlation of lesion volume and liver exposure

**Fig. 2:**
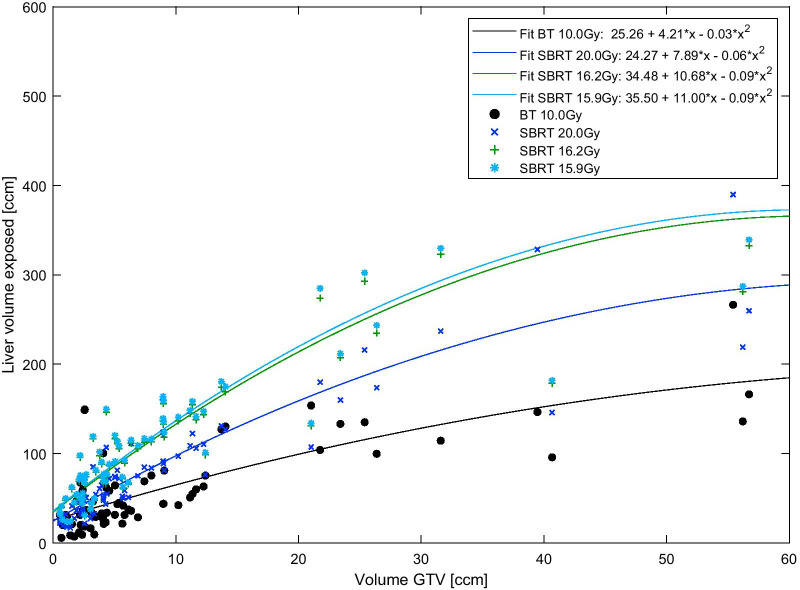
Correlation of lesion volume and liver exposure

Figure 2 shows the correlation of gross tumor volume (GTV) volume and liver exposure for all 71 lesion. The fit functions of the three SBRT doses considered are all steeper compared to the fit function of the corresponding BT dose. For a given GTV volume, SBRT liver exposure is, on average, larger than the BT liver exposure. Since the fit functions are diverging in the range of 0 ccm to about 40 ccm, the average difference in absolute exposed liver volume between SBRT and BT increases with GTV size.

### Conformity indices

**Fig. 3:**
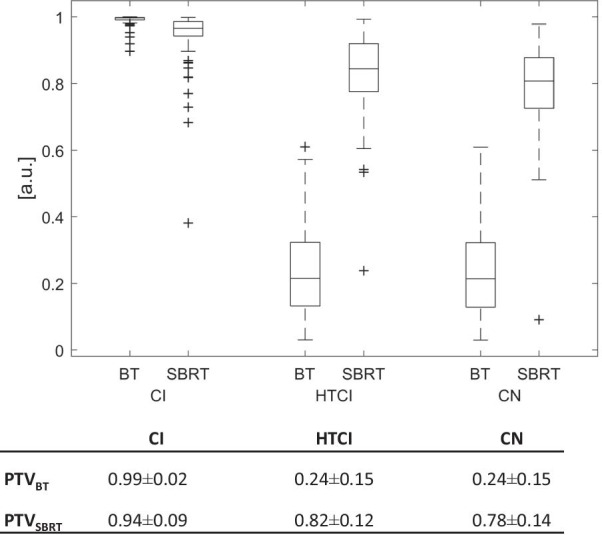
Conformity indices

To evaluate conformity, several indices were calculated. In HDR BT, the CI was 0.99 ± 0.02, the HTCI was calculated to be 0.24 ± 0.15, and the CN was 0.24 ± 0.15. For SBRT CI, HTCI, and CN were 0.94 ± 0.09, 0.82 ± 0.12, and 0.78 ± 0.14 respectively (Fig. 3.). The CI was significantly better for BT, while HTCI and CN were significantly better for SBRT (*p* < 0.001).

## Discussion

Radiation-based local ablative treatment options enrich the toolbox of LATs for patients with HCC not amendable for surgery. Stereotactic treatments allow to accurately deliver high doses of radiation to tumours, while sparing surrounding tissues, and are widely available with several indications such as stereotactic radiosurgery (SRS) of brain metastases [22, 23], SBRT of pulmonary metastases [24] or early-stage NSCLC [25]. SBRT in patients with HCC has been shown to be a safe and efficient approach with local control rates after 2 years of > 90% [20]. Currently, HDR BT of liver malignancies is emerging as an alternative to SBRT—however, the technique is currently only available in specialized centres and requires a close collaboration between interventional radiology and radiation oncology or a very experienced brachytherapy service.

This study evaluates clinically applied interstitial HDR BT cases in terms of normal liver sparing. A plan comparison was conducted to virtual SBRT plans using the original HDR BT planning CT. In the available literature comparing both modalities, usually CT datasets acquired for SBRT were used and compared to virtual brachytherapy plans [26, 27]. To the best of our knowledge only one other study by Hass et al. has so far used original BT planning CT data and compared to virtual SBRT plans [28]. They included HCC and liver metastases of various primary tumours and the prescribed single fraction doses were 15–20 Gy (D99.9%) for both, HDR BT and virtual SBRT. The study by Hass et al. has used a larger margin for SBRT PTV (5 mm lateral and 10 mm longitudinal) to account for the lack of information on tumour motion. They reported mean treatment volumes for brachytherapy PTV = 34.7 ccm and SBRT PTV = 73.2 ccm, which were considerably larger than treatment volumes in the present study with PTV_BT_ = 9.9 ccm and PTV_SBRT_ = 31.0 ccm. The study by Hass et al. did not find a significant difference for the liver exposure of 5 Gy in patients receiving a prescribed dose of 15 Gy, but a significant difference in patients treated with 20 Gy prescription dose. While these results give a good insight on the theoretical advantages of the respective radiation technique, a single fraction SBRT of liver malignancies with a prescribed dose of 15 Gy is not a clinically validated treatment regimen. Herfarth et al. reported on a phase I/II trial evaluating a cohort of 60 liver lesions treated with single fraction SBRT. Initially, a dose of 14 Gy was administered, but subsequently the dose was raised up to 26 Gy due to local failure rates [29].

**Fig. 4:**
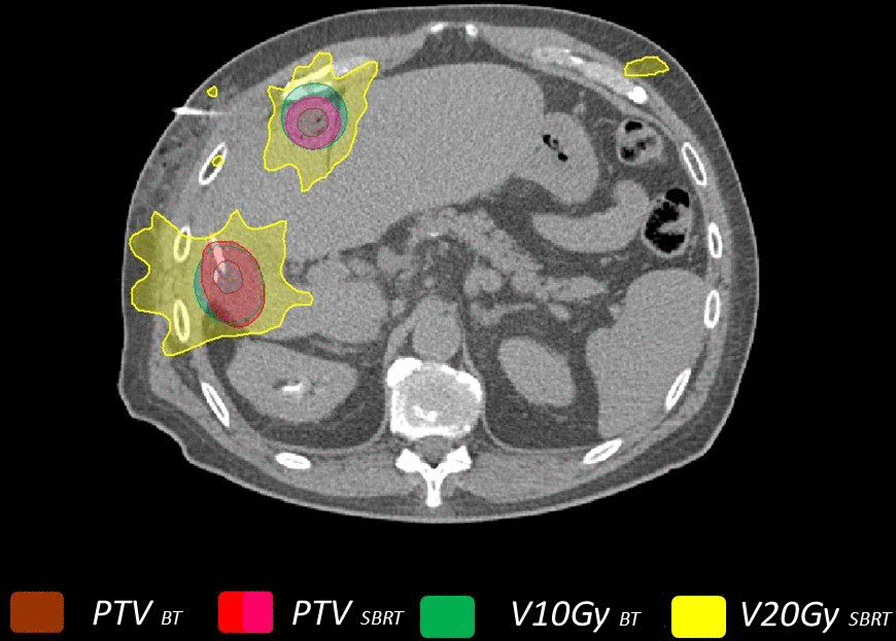
Liver exposure with BT (V10Gy, green) and SBRT (V20Gy, yellow) in a patient treated for two HCC lesions

The aim of the present study was to gain detailed insights into the dose exposure of uninvolved liver tissue in clinically applicable treatment regimens. We therefore compared a single fraction HDR BT to a fractionated SBRT. We acknowledged that a comparison of differing dose prescriptions and fractionation schemes is challenging. However we decided to compare well published and clinically applied treatment schedules rather than correlating doses which has been published by others. For SBRT we chose a prescribed dose at the lower end of the published data as we expected favourable liver exposure with this approach. We evaluated volumes with comparable EQD2 (α/β3, α/β2 for liver tissue), knowing that the LQ-model is limited in the dose range of the applied doses. Therefore, and more importantly, we performed a comparison of clinically relevant liver exposure. The definition of clinically relevant was based on the available literature on post interventional tissue changes visible in functional MRI using hepatocyte specific contrast media. We therefore compared Vliver10Gy_BT_ versus Vliver20Gy_SBRT_, which takes into account smaller volumes of liver exposure in SBRT than with an EQD2-based approach (Vliver15.9Gy_SBRT_ or Vliver16.2Gy_SBRT_). In our dataset we found that on average the Vliver10Gy_BT_ exposure in one single fraction delivered with HDR BT was smaller than the corresponding volume exposed to 20 Gy in fractionated SBRT (Vliver20Gy_SBRT_), suggesting an advantage of HDR BT for normal liver tissue sparing. An example is shown in Fig. 4. Notably, we decided to use an isotropical expansion by 6 mm to create a PTV_SBRT_ rather than mimicking an ITV concept. With this approach we did not take into account possible movements of the target lesion, which in a clinical scenario would require a larger PTV_SBRT_ volume. Therefore our results tend to underestimate the liver exposure in SBRT. Considering the fact that prognosis of patients with HCC is crucially depended on liver function sparing of uninvolved liver tissue needs to be prioritized. Traditionally the use of radiooncological treatment options in patients with HCC is limited because of the damage to uninvolved liver tissue by radiation causing radiation induced liver disease (RILD). Other LAT such as RFA and TACE are of today more successful in that respect. This demands improvements in radiooncological techniques in order to be competitive in a growing number of Patients with HCC.

Moreover, we determined the correlation between the GTV volume of the treated liver lesion and the radiation exposure to the liver. Figure 2 shows the advantage of HDR BT over SBRT in comparison of clinically relevant doses, and for EQD2 equivalent doses. The advantage was more pronounced for greater liver lesions which is seemingly in contrast to a recent planning study by Wust et al. [30] that described an advantage of HDR BT over different external beam radiotherapy techniques, especially for smaller lesions. In contrast to the study by Wust et al. where ideal catheter placement was assumed, the current study evaluates actually performed HDR BT treatments. In addition to the volume of the treated lesion, other factors such as the targeting accuracy of the catheter placement influences the liver exposure. Obviously, it is more challenging to puncture smaller lesions during fluoroscopic CT interventions and therefore a larger volume might be exposed to radiation in order to cover the entire lesion. Wust et al. based their conclusion on the evaluation of four different lesion sizes in one patient, which also showed smaller liver volumes exposed to 10 Gy for BT compared to VMAT or tomotherapy, even for the larger lesions (max. 75.9 ccm; larger than the largest lesion in our cohort). However, only the low dose liver exposure was reported by Wust et al. to be larger for BT compared to VMAT or tomotherapy for larger lesions. Low dose exposure was not assessed in our study. Overall, our results are not necessarily contradictious to the results provided by Wust et al., given the different study design and isodose values considered.

A secondary endpoint in this study was to compare dose coverage between HDR BT and SBRT plans. We compared a single dose HDR BT with a prescribed dose of 15 Gy to a fractionated SBRT with a prescribed dose of 37.5 Gy (65% isodose normalisation). Both treatment regimens have been published and have shown to achieve high rates of local control. For SBRT a D50 biologically equivalent dose ≥ 100 Gy assuming an α/β of 10 is required [31], while a study including 83 patients with a total of 140 HCC lesions treated with HDR BT found no significant dose dependence for doses between 15 and 25 Gy [32] suggesting that 15 Gy is sufficient to treat HCC lesions. More recently, it was hypothesised that in SBRT of liver metastases, reducing the PTV prescription dose while aiming at a high mean GTV dose reduced toxicity and still provided a good local control [33]. Therefore the mean dose D50 of the GTV might be of prognostic value. Accordingly we evaluated the D50 for HDR BT and SBRT plans. Due to the steep dose gradient that is inherent to HDR BT, a relatively high D50 was achieved in BT with 41.8 ± 8.1 Gy in a single fraction compared to 55.2 ± 2.3 Gy in SBRT in 3 fractions. While the net D50 in SBRT is still higher in this plan comparison, two important factors need to be considered. First, the absolute difference in D50 (41.8 ± 8.1 Gy vs. 55.2 ± 2.3 Gy) is smaller than the absolute difference in prescribed doses (15 Gy vs. 37.5 Gy), secondly, the D50 of 41.8 Gy in BT is administered in a single fraction, while the D50 of 55.2 Gy in SBRT is administered in three fractions. Although dose comparison using the LQ-model might not be valid at such high doses, the presented data might help explain why overall lower prescription doses can be applied in HDR BT versus SBRT to achieve similar rates of local control.

Furthermore, we evaluated the Conformity index (CI), Healthy tissue conformity index (HTCI) and Conformation number (CN) that were utilized by Milickovic et al. for comparison of HDR BT and SBRT plans [21]. An overview is given in Fig. 3. Our data show that the CI was high in both treatment modalities, HDR BT and SBRT, with 0.99 ± 0.02 versus 0.94 ± 0.09, respectively, with a value of 1.0 indicating perfect conformity. The HTCI measures the exposure of healthy liver tissue irradiation outside of the PTV with the prescribed dose—with a value of 1 indicating a perfect overlap. Therefore, a value of 0.82 ± 0.12 achieved by SBRT compared to 0.24 ± 0.15 obtained with HDR BT seems at first glance to be an advantage for SBRT. However, to interpret the HTCI one has to take into account the prescribed dose, which in this study differs considerably between the modalities. Therefore the absolute liver exposure remains favourable in BT over SBRT.

While homogeneity is considered important in brachytherapy treatment using multi-catheter techniques such as the treatment of breast and head and neck cancer, it is less important in the treatment of liver lesions. HDR BT in the liver usually targets lesions that are generally small and ideally few catheters are used. With the steep dose gradient that is inherent to brachytherapy, dose peaks within the tumor and small extremely hot areas in close proximity to the source are unavoidable and tolerated. Accordingly, the D2 for HDR BT was not reported, since such very high dose values calculated by the treatment planning system are of no validity.

The current study has some limitations due to its retrospective character. In particular, the virtual calculation of SBRT plans was performed without considering possible movements of the target volume. Consequently our results most likely underestimate the liver exposure in conventional liver SBRT. However, emerging techniques such as MR-guided radiotherapy [6] allow for more elaborate motion management (real time tracking) and therefore the assumed liver exposure in SBRT represents a more realistic estimation in view of technical advances.

## Conclusion

The results of this plan comparison support the assumption that the treatment of HCC lesions using HDR interstitial BT can be advantageous in respect of sparing of normal liver tissue as compared to SBRT, while providing excellent target conformity.

## Data Availability

The datasets used and/or analysed during the current study are available from the corresponding author on reasonable request.

## Abbreviations

HCC: hepatocellular carcinoma
LAT: locally ablative treatment
LT: liver transplantation
RFA: radiofrequency ablation
SBRT: stereotactic body radiation therapy
HDR BT: high-dose brachytherapy
ESMO: European Society for Medical Oncology
IV: intravenous
RILD: radiation induced liver disease
CT: computed tomography
MRI: magnetic resonance tomography
TPS: treatment planning software
OAR: organs at risk
CTV: clinical target volume
PTV: planning target volume
HIPO: hybrid inverse treatment planning optimization
ITV: internal target volume
CI: conformity index
PD: prescribed dose
V: volume
HTCI: healthy tissue conformity index
CN: conformation number
TACE: transcatheter arterial chemoembolization
GTV: gross tumor volume
SRS: stereotactic radiosurgery
VMAT: volumetric modulated arc therapy
